# Alcohol use disorder tied to development of chronic kidney disease: A nationwide database analysis

**DOI:** 10.1371/journal.pone.0203410

**Published:** 2018-09-06

**Authors:** Chi-syuan Pan, Teressa Reanne Ju, Chi Chan Lee, Yu-Pei Chen, Chung-Y. Hsu, Dong-Zong Hung, Wei-Kung Chen, I-Kuan Wang

**Affiliations:** 1 Department of Emergency Medicine, China Medical University Hospital, Taichung, Taiwan; 2 Mackay Memorial Hospital, Taipei, Taiwan; 3 Department of Critical Care, Oregon Health Science University Hospital, Portland, OR, United States of America; 4 Management Office for Health Data, China Medical University Hospital, Taichung, Taiwan; 5 Graduate Institute of Clinical Medical Science, China Medical University, Taichung, Taiwan; 6 Department of Nephrology, China Medical University Hospital, Taichung, Taiwan; University of Mississippi Medical Center, UNITED STATES

## Abstract

**Introduction:**

Alcohol use disorder (AUD) is a spectrum of high risk behaviors including alcohol abuse and dependence. Chronic kidney disease (CKD) is progressive loss of renal function for more or equal to 3 months or presence of any irreversible kidney damage. Common risk factors of CKD have been identified, but the impact of alcohol consumption on kidney function is controversial. The study aims to investigate the relationship between alcohol use disorder and CKD on a national scale.

**Methods:**

This retrospective cohort study was conducted using Taiwan’s National Health Insurance research database. Patients aged 20 years or older, without CKD and with the diagnosis of AUD (ICD-9-CM codes 303.X; 305.0, V113) from years 2000 to 2013 were enrolled. Control cohort was selected to match the demographics of the target population. Patients were followed until the end of 2013 or earlier if they developed CKD, died, or lost follow up. Baseline characteristics and comorbidities were identified for risk stratification.

**Results:**

We identified 11639 patients in the AUD cohort and 46556 patients in the control cohort. Compared to patients in the control cohort, those in the AUD group were more likely to have multiple comorbidities (p < 0.001 for all comorbidities). After adjustment of age, gender, baseline comorbidities, and nonsteroidal anti-inflammatory drug use, the diagnosis of AUD was associated with an increased risk of CKD development (aHR = 1.62, 95% CI, 1.46–1.81).

During the mean follow up periods of 6.47 (standard deviation (SD) = 3.80) years for the AUD cohort and 7.23 (SD = 3.75) years for the control cohort, the overall incidence density of CKD was significantly higher in patients with AUD than those in the control cohort (3.48 vs 6.51 per 1000 person-years, aHR = 1.68, 95% CI, 1.50–1.87). Kaplan-Meier analysis showed that the AUD cohort had a higher cumulative incidence of CKD than the control cohort (log-rank test, p value < 0.001). Patients with AUD had higher risks of CKD in all the stratified groups, except for the subgroup with age over 65 years old.

**Conclusion:**

Our study suggested that AUD was associated with an increased incidence of newly diagnosed CKD by nearly two folds. Young age, in particular, had a higher association between AUD and CKD. Considering the preventable nature of AUD, establishing effective health policies is imperative to reduce high-risk alcohol behaviors and thereby prevent alcohol-related kidney disease. Further prospective studies are warranted to further elucidate the causation of AUD on kidney function.

## Introduction

**Fig 1:**
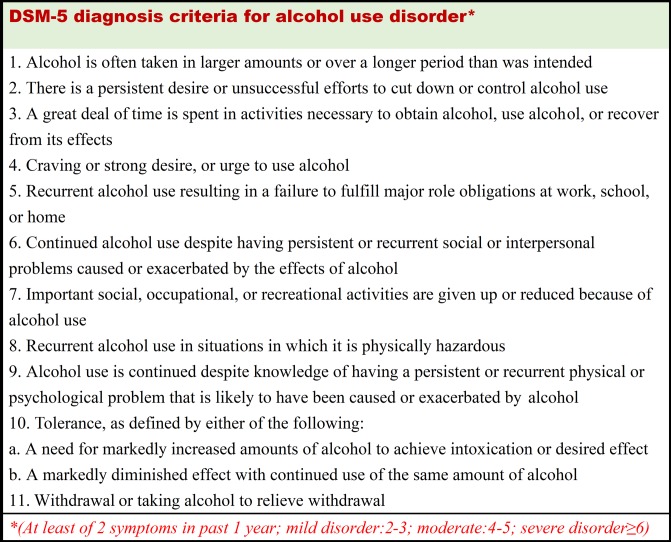
DSM-5 diagnosis criteria for alcohol use disorder.

Alcohol use disorder (AUD) is a spectrum of high risk behaviors including alcohol abuse and dependence. The Fifth edition of Diagnostic and Statistical Manual of Mental Disorders (DSM-5) defines AUD as the presence of at least 2 of 11 symptoms occurring within a 12-month period (Fig 1) [1]. International Classification of Diseases, Ninth Revision, Clinical Modification (ICD-9-CM), on the other hand, defines AUD as a composite of alcohol dependence syndrome, alcohol abuse and personal history of alcoholism [2]. According to the World Health Organization, the global prevalence rate of alcohol use disorder in adults was estimated to be 0% to 16% in 2004 [3]. AUD was associated with organ damage of liver, pancreas, heart and brain. In addition, it was tied to upper aerodigestive and colorectal cancers [4].

Chronic kidney disease (CKD) is defined as glomerular filtration rate (GFR) <60 ml/minute/1.73 m2 or urinary albumin excretion of ≥30 mg/day for more than 3 months [5]. The global prevalence of CKD was 11% to 13% [6]. The prevalence rate of CKD increased from 10.0% (1988–1994) to 13.1% (1999–2004) in the United States [7]. In Taiwan, the average prevalence rate of CKD was 11.9% from 1994 to 2006, and it was higher in patients with low socioeconomic status (19.87%) [8]. CKD is well known to increase mortality, incidence of cardiovascular diseases and hospitalization [9]. The two most common causes of CKD are diabetes mellitus (DM) nephropathy and hypertensive kidney disease. Other etiologies of CKD are glomerular disease, polycystic kidney disease [10], tubulointerstitial disease, obstructive nephropathy, nephrotoxic medication, contrast exposure [11]. and unknown cause [12].

Risk factors of CKD were well identified in previous literature, such as hypertension, smoking, nephrotoxic drugs and drug abuse [13, 14, 15]. However, the impact of alcohol consumption to kidney damage was contradictory. Some studies suggest that alcohol consumption was associated with an increased risk of CKD. In the population of Wisconsin, USA, heavy drinking, defined as more than four servings of alcohol per day, was tied to CKD. Joint exposure to both smoking and heavy drinking was associated with almost five times odds of developing CKD compared with their absence [16]. Another population-based case-control study in America suggested consumption of more than two alcoholic drinks per day was associated with an increased risk of kidney failure in the general population [17].

On the other hand, many articles revealed that alcohol consumption seemed to be a protective factor of CKD development and the effect was directly proportional to the amount of alcohol use. In a prospective study [18], healthy men who consumed alcohol at least seven drinks weekly had lower odds of renal dysfunction. This effect was not transferred to the population who consumed alcohol less than seven drinks weekly. White et al. did a population-based prospective study in Australia and revealed that alcohol intake of more than 30 grams per day was associated with a reduced risk of estimated GFR <60 mL/min/1.73 m2 [19]. Kronborg et al. conducted a seven-year longitudinal follow up study in Norway, and the results showed that alcohol consumption of more than 72 grams per week was associated with higher estimated GFR in men but not in women [20].

Similar results were observed in Eastern Asia populations. A multivariable cross-sectional study suggested alcohol consumption was negatively associated with the presence of stage 3 CKD in Taiwanese men, particularly in patients with frequent drinking behaviors [21]. In Japanese men of good health, an inverse association was found between the frequency of drinking alcohol and CKD [22]. Interestingly, another Japanese study showed that while alcohol consumption of any amount was associated with lower prevalence of CKD, less than 19 grams per day of alcohol use was associated with lower risk of proteinuria [23].

Given the contradiction of current literature, the present study aims to investigate the relationship between AUD and CKD on a larger scale by analyzing data originating from a national database.

## Methods

### Data source

This study used data from the National Health Insurance research database (NHIRD), which included health care information of 99% of Taiwanese population since 1995. CKD ascertainment in NHIRD database using ICD-9-CM code was validated in a previous published article [24, 25, 26]. In 2000, The Longitudinal Health Insurance Database randomly sampled 1 million individuals from the beneficiaries of the NHIRD and formulated a LHID2000 database. LHID2000 database contained detailed claimed data of beneficiaries such as ambulatory claims, inpatient claims and drug prescription. Information regarding personal identification was encrypted before release. In our study, all diagnoses and disease definitions were specified using the ICD-9-CM codes. The study was approved by the Research Ethics Committee of China Medical University and Hospital in Taiwan (CMUH-104-REC2-115).

#### Study design and population

In this retrospective cohort study, we enrolled patients with 20 years of age or older who were diagnosed with AUD (ICD-9-CM codes 303.X, 305.0, V113) from 2000 to 2013 and created the study cohort. To match the baseline characteristics of patients from the study cohort, we randomly selected four persons without AUD with frequency matching for gender, age, and the index year, which was the year of index date, to form the control cohort. The follow-up period started at the date of establishing diagnosis of AUD and ended on Dec 31, 2013 or earlier if patients developed CKD, death, withdrawal from health insurance, or loss to follow-up. Data was censored if patients died during the follow-up period. Competing risk analysis was not performed, since mortality rate of study population was only 5.7%. Patients with an established diagnosis of CKD (ICD-9-CM: 585) were excluded. Individuals with missing data, such as age and gender, were not enrolled in this study.

### Outcome and comorbidity

Age was stratified and categorized into three subgroups: elderly (age ≥ 65 years), middle-aged (age ≥ 50 and < 64 years) and young-aged (age < 50 years). The baseline comorbidities were selected for risk adjustment if they were known to be associated with CKD development. Atrial fibrillation (AF; ICD-9-CM codes 427.31), hypertension (ICD-9-CM codes 401–405), hyperlipidemia (ICD-9-CM codes 272), cerebral vascular accident (CVA; ICD-9-CM codes 430–438), chronic obstructive pulmonary disease (COPD; ICD-9-CM codes 491, 492, 496), cirrhosis (ICD-9-CM codes 571), coronary artery disease (CAD; ICD-9-CM codes 410–414), obesity (ICD-9-CM codes 278), asthma (ICD-9-CM codes 493), heart failure (ICD-9-CM codes 428) and diabetes (ICD-9-CM codes 250) were comorbidities identified for risk stratification. Non-steroid anti-inflammatory drugs (NSAID) use was defined as exposure to any types or dose of NSAID over 30 days in the follow-up duration. The data regarding NSAID use originated from patients’ health records of clinics or hospitals.

### Statistical analysis

We used the Chi-Square test to compare patient’s demographics and baseline comorbidities between patients with alcohol use disorder and the control cohort. Independent Student’s t test was used for analyzing continuous variables. We estimated the hazard ratios (HRs) and 95% confidence intervals (CIs) for CKD by univariate and multivariate Cox proportional-hazards regression models with stratification based on gender, age, and comorbidity. We adjusted the variables of age, gender, comorbidities and NSAID use in the multivariable models. We used Kaplan-Meier method to calculate the cumulative incidence of CKD. The survival curves of patients with alcohol use disorder and control cohort were compared using a log-rank test. The statistical analyses were conducted using SAS System for Windows, Version 9.4. All significant thresholds were set at two-sided *P* value less than 0.05.

## Results

We identified 11639 patients in the AUD cohort and 46556 patients in the non-alcohol use disorder cohort.

Table 1 demonstrated the demographics and comorbidity of AUD patients and the control cohort. Both cohorts had similar distributions in age and gender (Table 1). Compared to patients in the control cohort, those in the AUD cohort were more likely to have AF, hypertension, hyperlipidemia, CVA, COPD, cirrhosis, CAD, asthma, HF, and DM (*p* < 0.001) for all comorbidities. There are greater number of patients who used NSAID in the AUD cohort as compared to those in the control cohort (*p* < 0.001).

**Table 1:** Comparison of demographics and comorbidity between alcohol use disorder patients and the control cohort.

	Alcohol use disorder				
	No (N = 46556)		Yes (N = 11639)		
	n(%)		n(%)		*p*-value
Age, years					0.99
<50	33972 (72.97)		8493 (72.97)		
50–65	9864 (21.19)		2466 (21.19)		
>65	2720 (5.84)		680 (5.84)		
Mean (SD) †	42.88 (12.82)		42.90 (12.75)		0.8315
Gender					0.99
Female	10432 (22.41)		2608 (22.41)		
Male	36124 (77.59)		9031 (77.59)		
Comorbidity					
Atrial fibrillation	219 (0.47)		111 (0.95)		<0.0001*
Hypertension	8725 (18.74)		3390 (29.13)		<0.0001*
Hyperlipidemia	8369 (17.98)		2773 (23.83)		<0.0001*
CVA	3199 (6.87)		1488 (12.78)		<0.0001*
COPD	5154 (11.07)		1762 (15.14)		<0.0001*
Cirrhosis	12450 (26.74)		5528 (47.5)		<0.0001*
CAD	4916 (10.56)		1851 (15.9)		<0.0001*
Obesity	493 (1.06)		129 (1.11)		0.6429
Asthma	3694 (7.93)		1235 (10.61)		<0.0001*
Heart failure	695 (1.49)		346 (2.97)		<0.0001*
Diabetes	2376 (5.1)		1007 (8.65)		<0.0001*
NSAID use > 30 days	33320 (71.57)		9817 (84.35)		<0.0001*

Chi-square test examined categorical data

†T-test examined continuous data

*Statistical significance

SD, standard deviation; CVA, cerebral vascular disease; COPD, chronic obstructive pulmonary disease; CAD, coronary artery disease; NSAID, nonsteroidal anti-inflammatory drugs

Table 2 revealed the crude and adjusted HR (aHR) of risk factors for CKD of the entire study population. Male gender (aHR = 1.62, 95% CI, 1.46–1.81) and age over 65 years old (aHR = 3.85, 95% CI, 3.31–4.48) were associated with an increased risk of developing CKD. Based on the crude model, participants with any of the comorbidities had a higher risk of CKD (*p* < 0.001). After risk factors were adjusted, patients with AUD still had a higher risk of CKD (crude HR = 1.91, 95% CI, 1.72–2.13; aHR = 1.62, 95% CI, 1.46–1.81). Other comorbidities tied to CKD development after risk factors adjustment were hypertension, hyperlipidemia, CVA, cirrhosis, heart failure and DM. In contrast, NSAID use for more than 30 days during the follow-up period was not associated with higher risks of developing CKD.

**Table 2:** The incidences and risk factors for developing chronic kidney disease of study population.

Variable	Event	PY	Rate^#^	Crude HR (95% CI)	Adjusted HR^†^ (95% CI)
Alcohol use disorder					
No	1170	336545	3.48	1.00	1.00
Yes	490	75264	6.51	1.91 (1.72, 2.13)***	1.62 (1.46, 1.81)***
Age, years					
<50	636	312961	2.03	1.00	1.00
50–65	656	79613	8.24	4.21 (3.78, 4.70)***	2.14 (1.90, 2.42)***
>65	368	19234	19.13	10.11 (8.88,11.50)***	3.85 (3.31, 4.48)***
Gender					
Female	190	93754	2.03	1.00	1.00
Male	1470	318055	4.62	2.28 (1.96, 2.65)***	1.57 (1.35, 1.82)***
Comorbidity					
Atrial fibrillation					
No	1631	410147	3.98	1.00	1.00
Yes	29	1662	17.45	4.81 (3.33, 6.95)***	1.18 (0.83, 1.69)
Hypertension					
No	758	337419	2.25	1.00	1.00
Yes	902	74390	12.13	5.66 (5.14, 6.23)***	1.96 (1.74, 2.21)***
Hyperlipidemia					
No	966	344269	2.81	1.00	1.00
Yes	694	67540	10.28	3.87 (3.51, 4.27)***	1.32 (1.18, 1.48)***
CVA					
No	1288	382657	3.37	1.00	1
Yes	372	29152	12.76	3.90 (3.48, 4.38)***	1.17 (1.03, 1.33)*
COPD					
No	1310	370215	3.54	1.00	1.00
Yes	350	41594	8.41	2.52 (2.24, 2.83)***	0.96 (0.84, 1.10)
Cirrhosis					
No	876	295695	2.96	1.00	1.00
Yes	784	116114	6.75	2.36 (2.14, 2.60)***	1.22 (1.10, 1.36)***
CAD					
No	1176	369741	3.18	1.00	1.00
Yes	484	42067	11.51	3.78 (3.40, 4.20)***	1.05 (0.93, 1.19)
Obesity					
No	1641	408541	4.02	1.00	1.00
Yes	19	3268	5.81	1.58 (1.01, 2.49)***	0.79 (0.50, 1.26)
Asthma					
No	1434	382975	3.74	1.00	1.00
Yes	226	28834	7.84	2.22 (1.93, 2.56)***	1.09 (0.94, 1.27)
Heart failure					
No	1549	406435	3.81	1.00	1.00
Yes	111	5374	20.66	5.87 (4.84, 7.12)***	1.33 (1.08, 1.64)**
Diabetes					
No	1231	393627	3.13	1.00	1.00
Yes	425	18341	23.17	7.99 (7.15, 8.93)***	3.05 (2.70, 3.44)***
NSAID use >30 days					
No	273	103878	2.63	1.00	1.00
Yes	1417	307876	4.6	1.75 (1.53, 1.99)***	0.97 (0.84, 1.11)

CI, confidence interval; HR, hazard ratio; PY, person-years; CVA, cerebral vascular disease; COPD, chronic obstructive pulmonary disease; CAD, coronary artery disease; NSAID, nonsteroidal anti-inflammatory drugs

^#^Incidence rate per 1,000 person-years

^†^Model was adjusted for age, sex, comorbidities and NSAID use by using Cox proportional hazards regression

*p < 0.05

**p < 0.01

***p < 0.001

Table 3 compared the incidence and adjusted hazard ratio of developing CKD between AUD cohort and control cohort. The data was further stratified by gender, age and the presence of comorbidity. The overall incidence density of CKD was significantly higher in patients with AUD than those in the control cohort (6.51 versus 3.48 per 1000 person-years, crude HR = 1.91, 95% CI, 1.72–2.13) with an aHR of 1.68 (95% CI, 1.50–1.87). In the subgroup analysis, patients with AUD had higher risks of CKD in all the stratified groups in comparison to those from control cohort, except for the subgroup with age over 65 years old (aHR = 0.97, 95% CI, 0.84–1.11).

**Table 3:** Incidence and adjusted hazard ratio of developing chronic kidney disease and person-years by gender, age and comorbidity for patients with alcohol use disorder in comparison to the control cohort.

	Alcohol use disorder						Compared to Control	
	No			Yes				
Variables	Events	PY	Rate^#^	Events	PY	Rate^#^	Crude HR*	Adjusted HR^†^
							(95% CI)	(95% CI)
All	1170	336545	3.48	490	75264	6.51	1.91 (1.72, 2.13)***	1.68 (1.50, 1.87)***
Gender								
Female	125	75693	1.65	65	18061	3.60	2.20 (1.63, 2.96)***	2.05 (1.50, 2.82)***
Male	1045	260852	4.01	425	57203	7.43	1.90 (1.70, 2.13)***	1.64 (1.46, 1.84)***
Age, years								
<50	398	255123	1.56	238	57838	4.11	2.69 (2.29, 3.16)***	1.94 (1.64, 2.30)***
50–65	474	65593	7.23	182	14020	12.98	1.89 (1.59, 2.24)***	1.48 (1.24, 1.76)***
>65	298	15829	18.83	70	3405	20.56	1.13 (0.87, 1.47)	1.08 (0.83, 1.40)
Comorbidity^§^								
No	259	185904	1.39	62	25918	2.39	1.77 (1.35, 2.34)***	2.28 (1.70, 3.00)***
Yes	911	150641	6.05	428	49346	8.67	1.45 (1.29, 1.63)***	1.66 (1.48, 1.86)***

PY, person-years; Rate^#^, incidence rate, per 1,000 person-years; Crude HR*: relative hazard ratio

Adjusted HR^†^: adjusted hazard ratio controlling for age, sex and comorbidities

Comorbidity^§^: Patients with any one of the comorbidities atrial fibrillation, hypertension, hyperlipidemia, CVA, COPD, cirrhosis, CAD, obesity, asthma and heart failure were classified as the comorbidity group

*p<0.05

**p<0.01

***p<0.001

**Fig 2:**
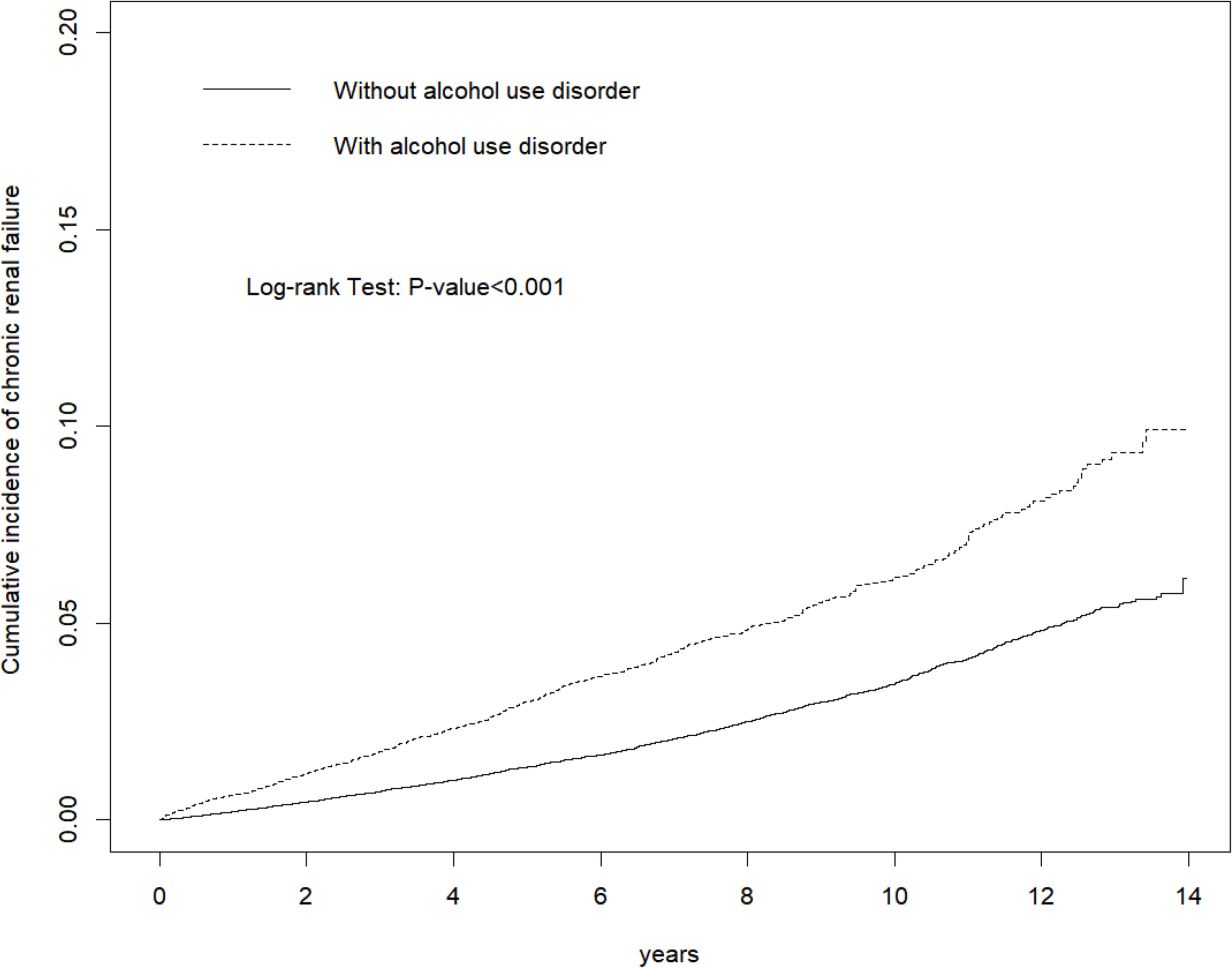
Cummulative incidence of chronic kidney disease in patients with alcohol use disorder and control cohort.

The range of follow-up period for both cohorts was approximately 14 years. The median of follow-up period for AUD cohort and control cohort was 6.25 and 7.29 years respectively. During the mean follow up periods of 6.47 (standard deviation (SD) = 3.80) years for the AUD cohort and 7.23 (SD = 3.75) years for the control cohort, Kaplan-Meier analysis showed that the AUD cohort had a higher cumulative incidence of CKD than control cohort (log-rank test, *p* < 0.001) (Fig 2).

## Discussion

The present study demonstrated AUD was tied to chronic kidney disease, even after adjustment of gender, age, comorbidities and NSAID use. The cumulative incidence of CKD was higher in patients with AUD during longitudinal follow-up.

Previous literature had shown that alcohol consumption was associated with multiple adverse outcomes [4, 16, 27]. Our study coincided with the findings of previous studies, that AUD was strongly associated with multiple chronic conditions (Table 1). We also noted that in our study AUD was linked to increased NSAID use. While the exact mechanism of the association was unclear, alcohol use was known to increase the risk of gout by two folds [28]. This, in turn, may be attributed to increased NSAID use in patients with alcohol consumption.

Chronic kidney disease is defined as abnormalities of kidney structures or functions and present for more than 3 months [10]. Common risk factors of CKD were recognized in previous literatures, such as age, gender, cardiovascular diseases, and diabetes mellitus [29]. In our study, age more than 65 years old was the strongest risk factor of CKD. Other identified risk factors of CKD in our study included male and baseline comorbidities, such as diabetes, hypertension, atrial fibrillation, hyperlipidemia, coronary artery disease, heart failure and cirrhosis. As for the primary interest of our study, alcohol use disorder was associated with an increased risk of CKD by nearly two folds after adjustment of age, gender, NSAID use, and baseline comorbidities. Similar to results from previous study, alcohol consumption was not associated with developing CKD particularly in elderly population [30]. This phenomenon suggests that in elderly population, aging probably plays a more dominant role in CKD development as compared to AUD. Further randomized trial is needed to elaborate the causality. On the other hand, it is worth mentioning that young-aged population with AUD had a much higher risk of developing CKD (adjusted HR = 1.94, 95% CI, 1.64–2.30). Given the current alcohol epidemic among youth worldwide, this finding highlights the importance of reducing high-risk alcohol behaviors in this targeted population [31].

Our study demonstrated the association between alcohol use disorder and CKD development, which was contradictory to many of published studies. The discrepant results among studies may be attributed to a number of reasons. First, the reliability of exposure ascertainment was a major limitation of published studies. Most studies used self-reports of alcohol consumption in their data collection, and the accuracy of self-reported data were affected by recall bias, design of questionnaires, respondent characteristics, and the social norm of alcohol use [32]. A prospective study had shown that recall bias existed even when respondents answered questionnaires within seven days of last alcohol use [33]. Second, primary outcome of most studies was estimated GFR, which was calculated through serum creatinine levels. Different equations of GFR calculation (i.e. Cockcroft-Gault Formula or Modification of Diet in Renal Disease Study equation) may result in different estimated GFR despite using the same serum creatinine value. Third, surveillance bias may exist since patients with alcohol use disorder likely receive more frequent kidney function checkup. The scale of surveillance bias is determined by health care accessibility, health equality and public health policies of individual countries. Fourth, serum creatinine levels could be largely affected by patients’ muscle mass, liver function, gender and age [34]. In cirrhotic patients with heavy alcohol consumption, impaired creatinine synthesis due to liver dysfunction, and loss of muscle mass due to chronic nutritional deficiencies led to spuriously low serum creatinine levels. As a result, kidney function was generally overestimated in cirrhotic patients. Cystatin C, a novel serum biomarker that rises in the setting of kidney injury, may be a better surrogate marker for assessing kidney function in patients with liver impairment [35]. In comparison to serum creatinine, Cystatin C was less affected by age, gender, and muscle mass [36, 37]. It potentially offers better accuracy in assessing GFR [38] and could be utilized in future studies involving patients with AUD. Last, different alcoholic beverages may affect kidney function distinctively due to their non-alcoholic contents. For example, polyphenol contents in wine and beer had strong antioxidant properties and carry protective effects against vascular aging [39]. Previous randomized cross-over trial had shown moderate consumption of red wine, but not gin, provided additional antioxidant effects on cells [40]. In diabetic mice, polyphenols protect kidney damage through suppressing nuclear factor-kappa B [41]. Future human studies are needed to determine the impact of different alcoholic beverages on kidney function.

Our study had certain strengths. Compared to majority of studies, we used disease-specific diagnosis codes rather than self-reported questionnaires to identify targeted populations. Therefore, we were able to avoid the nature of recall bias of self-reported studies. Moreover, we only included patients with no established diagnosis of CKD. Therefore, through a 7-year follow up we could clearly acquire the cumulative incidence of newly developed CKD and identify associated risk factors, which was not feasible with cross-sectional studies. Last, the scale of the study and the long duration of follow-up period made the result of study plausible.

Our study had several limitations. First, this study could only identify those patients with established diagnosis of AUD, thus patients with light or moderate amount of alcohol consumption or who were not evaluated by physicians were excluded from this study. Second, dose-response relationship cannot be assessed in our study. The diagnostic criteria of AUD primarily refer to the constellation of symptoms from alcohol use rather than the amount of alcohol consumption. However, based on the study conducted by Saunders et al. [42], we assume the amount of alcohol drinking in AUD is more than 60 grams per day for men and 40 grams per day for women. Third, patients with missing data, while accounted for small portions of the whole dataset, was not included in our study cohort. Fourth, the data from the NHIRD database didn’t include smoking history and socioeconomic status, which could be potential confounders. Last, we did not assess the specific types or dose-outcome relationship of NSAID use and did not include the history of using other nephrotoxic drugs in this study. History of consuming energy drinks was not available in this dataset as well.

## Conclusion

Our study demonstrated that alcohol use disorder was tied to newly diagnosed CKD by nearly two folds. Young-aged, in particular, had a higher association between alcohol use disorder and CKD. Considering the preventable nature of high risk drinking, establishing effective public health policies is imperative to reduce high-risk alcohol behaviors and thereby alcohol-related kidney disease. Further prospective studies, preferably with well documented alcohol doses and diagnosis of AUD, are warranted to elucidate the causation of alcohol use disorder on kidney function. Novel biomarkers, such as cystatin C, may be considered as a surrogate marker for assessing kidney injury in patients with cirrhosis and AUD.
